# Rates of Cognitive Decline in 100 Patients With Alzheimer Disease

**DOI:** 10.31486/toj.21.0084

**Published:** 2022

**Authors:** Monica Miyakawa-Liu, Amy K. Feehan, Shannon Pai, Julia Garcia-Diaz

**Affiliations:** ^1^ Department of Pathology, Stanford University, Stanford, CT; ^2^ Department of Infectious Diseases, Ochsner Clinic Foundation, New Orleans, LA; ^3^ The University of Queensland Medical School, Ochsner Clinical School, New Orleans, LA

**Keywords:** *Alzheimer disease*, *cognitive dysfunction*, *dementia*, *mental status and dementia tests*, *neuropsychological tests*

## Abstract

**Background:** In the state of Louisiana, the prevalence of Alzheimer disease (AD) is projected to increase 26.4% by 2025 because of the rapidly increasing geriatric population. While significant research is available on risk factors for developing AD, less data are available regarding AD progression and the rate of change among patients with the disease. To date, no research has established the baseline cognitive decline of patients with AD residing in New Orleans, Louisiana.

**Methods:** We evaluated 100 patients in the Ochsner Health system from September 2013 to December 2019 who had a diagnosis of AD and repeated Mini-Mental State Examination (MMSE) or Montreal Cognitive Assessment (MoCA) scores to determine annual rates of decline. Associated variables that were analyzed included race, age at diagnosis, social factors, and comorbidities.

**Results:** The average annual rates of decline for MMSE and MoCA scores were 2.43 (SD 2.82) points and 2.39 (SD 1.88) points, respectively. Our results were significant for a faster rate of decline in MMSE scores among smokers (3.50 points, SD 3.20) vs nonsmokers (1.54 points, SD 2.07). We found no significant difference in MoCA scores for smokers vs nonsmokers, in addition to other demographic and lifestyle variables.

**Conclusion:** The rate of decline seen in an urban population of patients with AD is lower than the average rate of decline reported in the literature, a finding that can help inform future interventional studies that use rate of decline as a primary outcome.

## INTRODUCTION

In 2015, Alzheimer disease (AD) was identified as the sixth leading cause of death in Louisiana, which also had the tenth highest AD death rate in America in the same year.^1^ In Louisiana alone, the prevalence of the disease is projected to increase 26.4% by 2025 because of the rapidly increasing geriatric population.^1^ AD is the most common cause of dementia and poses a significant health care cost and burden to families and caregivers. The lifetime cost of care for an American living with dementia was estimated to be $341,840 in 2017.^1^ AD is associated with numerous polymorphisms that are often geographic-specific.^2,3^ One example is the low-density lipoprotein receptor-related protein 1, which is a candidate gene for AD pathogenesis and has a decreasing prevalence from the northern to the southern regions of Europe.^3^ While significant research is available on risk factors for developing AD, less data are available regarding AD progression and the rate of change among patients with the disease.

No definitive test is available for diagnosis of AD, but screening tools such as the Mini-Mental State Examination (MMSE) and the Montreal Cognitive Assessment (MoCA) can be used to assess different cognitive domains—visuospatial, language, concentration, memory recall, and orientation—and these assessments can be used as reference points for individual cognitive decline. Both tests have a maximum of 30 points; scores ≥26 are considered normal. The MMSE is one of the most common ways of assessing the progression of AD at follow-up visits. The Han et al meta-analysis showed that the average annual rate of decline in the MMSE score is 3.3 points.^4^ However, significant heterogeneity exists among studies, and the rates of decline vary widely among individuals of different demographics.^5^ Many additional factors likely contribute to the variation in disease progression, including cognitive reserve, genetics, comorbidities, and medical and social support.^5^ Even the effect of comorbidities on disease progression is controversial. For example, diabetes mellitus was associated with both fast and slow rates of cognitive decline in different articles.^5,6^ In terms of sex, AD has disproportionately affected more women than men; however, the evidence as to whether sex influences the rate of decline is complicated.^7,8^ The presence of the APOE4 allele has been reported to increase the risk of AD in women,^9^ yet other studies support estrogen as a neuroprotectant.^10^

When comparing the rates of cognitive decline between races, the Chicago Health and Aging Project (CHAP) revealed that a higher proportion of African Americans had rapid and moderate decline in global cognition than European Americans (7% vs 5% for rapid decline and 20% vs 15% for moderate decline, respectively).^11,12^ Based on 4 tests, including the MMSE, the CHAP study used a composite cognitive score to calculate and categorize the rate of decline.^2^ In a separate Italian study, additional factors such as younger age at diagnosis, significant family history of AD, or more years of education were also associated with a higher rate of decline.^7^ However, in their meta-analysis, Han et al concluded that many of these covariants were not statistically significant in explaining the variability in decline rates.^4^

No studies have evaluated the rate of decline in patients with AD in New Orleans, Louisiana. Establishing a registry of the current population of patients with AD at Ochsner Medical Center-New Orleans and their demographics could help inform future studies and power analyses for interventional studies that use cognitive decline as a primary outcome. Overall, the literature characterizes AD as a disease that has significant variability in different geographic locations and patient populations, and this study is the first, to our knowledge, to describe the rate of decline in patients with AD in New Orleans.

## METHODS

Institutional review board approval was obtained (2019.384). Medical record numbers of patients with a diagnosis of dementia—*International Classification of Diseases*-10 codes for vascular dementia without behavioral disturbance (F01.50), unspecified dementia without behavioral disturbance (F03.90), unspecified dementia with behavioral disturbance (F03.91), Alzheimer disease with early onset (G30.0), Alzheimer disease with late onset (G30.1), Alzheimer disease, unspecified (G30.9), or mild cognitive impairment, so stated (G31.84)—and who were evaluated by either neurology or neuropsychiatry at the Ochsner Medical Center-New Orleans campus were extracted via Slicer Dicer, the search tool available in the Epic electronic medical record (Epic Systems Corporation). Cognitive screening tests were administered predominantly in the outpatient clinical setting by a board-certified neurologist, psychiatrist, or allied health care professional. The report was generated on December 13, 2019, and included 354 patients since September 2013.

Inclusion criteria required that individuals have at least 2 MMSE or MoCA scores in clinical visits separated by at least 6 months and a clinical notes statement that a diagnosis of AD was suspected or confirmed. Participants whose MMSE or MoCA scores were >26 were excluded because these patients would not be considered cognitively impaired. One hundred patients met the inclusion criteria and were included in this analysis. Differences in mean annual change of MMSE or MoCA scores by demographic and lifestyle factors were assessed by *t* test. Differences were deemed significant at *P*<0.05.

## RESULTS

Of the 100 patients with AD, 59% were female and 41% were male. The average age at diagnosis was 74.75 years, and 73% of patients self-identified as White. Sixty-one percent of patients were married, and 28% were widowed. The average MMSE score was 22.63 points (SD 5.77), and the average annual decline was 2.43 points (SD 2.82). The average MoCA score was 18.19 points (SD 5.56), and the average annual decline was 2.39 points (SD 1.88). Notable comorbidities for the population included smoking (40%), alcohol use (36%), and major depressive disorder (34%) (Table 1).

**Table 1.** Clinical and Demographic Data for All Patients

Variable	Patients with Alzheimer Disease, n=100
Mini-Mental State Examination score, mean (SD)	22.63 (5.77)
Montreal Cognitive Assessment score, mean (SD)	18.19 (5.56)
Decline in Mini-Mental State Examination score per year, mean (SD)	2.43 (2.82)
Decline in Montreal Cognitive Assessment score per year, mean (SD)	2.39 (1.88)
Age at diagnosis, years, mean (SD)	74.75 (7.67)
Male	41 (41)
Marital status	
Married	61 (61)
Widowed	28 (28)
Single	5 (5)
Divorced	4 (4)
Other	2 (2)
Race	
White	73 (73)
Nonwhite (Black, Hispanic)	27 (27)
Vascular risk factors	
Diabetes mellitus	29 (29)
Cerebrovascular accident	13 (13)
Smoking	40 (40)
Alcohol	36 (36)
Hypothyroidism	19 (19)
Major depressive disorder	34 (34)

Note: Data are presented as n (%) unless otherwise indicated.

In the assessment of annual rate of decline in MMSE or MoCA scores between different populations, the only variable with a significant difference was smokers vs nonsmokers in MMSE scores (Figure). Other variables such as sex, race, depression, diabetes, history of cerebrovascular accident, hypothyroidism, alcohol usage, marital status, and age at diagnosis did not reveal significant changes in annual cognitive decline in this cohort (Table 2).

**Figure.**
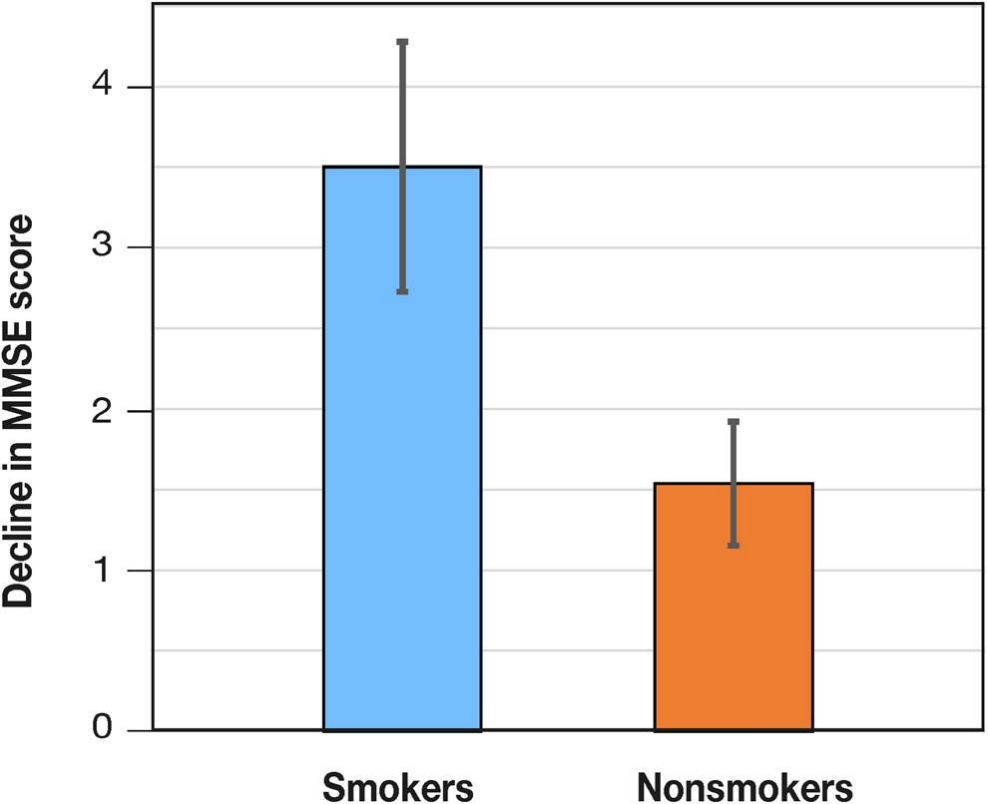
Average annual decline in Mini-Mental State Examination (MMSE) scores in smokers vs nonsmokers.

**Table 2.** *T* Test Results for Two Independent Means to Assess Mini-Mental State Examination (MMSE) and Montreal Cognitive Assessment (MoCA) Score Changes Over Time in Patients With Alzheimer Disease

Variable	Decline in MMSE Score	Decline in MoCA Score
Sex		
Male	2.26 (2.67)	2.06 (1.82)
Female	2.25 (2.79)	2.43 (1.89)
*T* Value	0.02	0.77
*P* Value	0.49	0.22
Race		
White	2.27 (2.92)	2.43 (1.94)
Nonwhite (Black, Hispanic)	2.92 (2.35)	2.30 (1.77)
*T* Value	0.65	0.25
*P* Value	0.26	0.40
Marital status		
Married	2.31 (2.98)	2.29 (1.79)
Widowed	2.01 (2.20)	2.83 (1.87)
*T* Value	0.37	1.02
*P* Value	0.36	0.16
Age		
<75 years	2.88 (3.43)	2.18 (2.15)
≥75 years	2.01 (2.11)	2.56 (1.64)
*T* Value	1.05	0.78
*P* Value	0.15	0.22
Smoking		
Smoker	3.50 (3.20)	2.07 (1.72)
Nonsmoker	1.54 (2.07)	2.58 (1.96)
*T* Value	2.53	1.03
*P* Value	0.007	0.15
Alcohol		
Alcohol	2.96 (3.46)	1.98 (1.77)
No alcohol	1.94 (1.73)	2.64 (1.89)
*T* Value	1.32	1.34
*P* Value	0.097	0.093
Diabetes mellitus		
With	3.36 (3.33)	1.85 (1.59)
Without	2.10 (2.60)	2.58 (1.97)
*T* Value	1.34	1.34
*P* Value	0.09	0.09
Cerebrovascular accident		
With	2.48 (1.96)	2.50 (1.90)
Without	2.31 (2.87)	2.39 (1.88)
*T* Value	0.14	0.15
*P* Value	0.44	0.44
Hypothyroidism		
With	2.24 (2.04)	2.45 (1.41)
Without	2.24 (2.76)	2.62 (2.46)
*T* Value	0.002	0.23
*P* Value	0.50	0.41
Major depressive disorder		
With	1.61 (1.94)	2.22 (1.96)
Without	2.60 (3.01)	2.52 (1.84)
*T* Value	1.16	0.58
*P* Value	0.13	0.28

Note: Data other than *T* and *P* values are presented as n (%).

## DISCUSSION

Our study found an average annual decrease in the MMSE (2.43 points) and MoCA (2.39 points) scores that were almost a full point smaller than reported in the Han et al meta-analysis (MMSE score, 3.3 points).^4^ An important point is that meta-analyses capture a wide variety of patients and their socioeconomic status, access to care, and genetic variants. Studies analyzing patients from a single community population have shown smaller annual decline rates in MMSE scores, including a study from Shanghai, China, in which the decline was as low as 1.52 points.^13^ The patients in our specific population were all insured and followed by specialists in neurology and psychiatry. Furthermore, the majority were married and were assumed to have familial support, which could explain the lower rate of decline when compared to the Han et al meta-analysis.^4^ Recent evidence (2018) suggests that social isolation and its role in epigenetics can increase the risk of or worsen cognitive decline in AD.^14^

In our study, the MoCA average score was lower than the MMSE average (18.19 and 22.63 points, respectively). While both the MMSE and MoCA are reliable screening tools for a diagnosis of AD, the 2 tests were developed under different contexts. MMSE was developed in 1975 as a faster way to quantitatively assess cognitive performance compared to previously established cognitive testing.^15^ The MoCA was formulated 30 years later for differentiating mild cognitive impairment from healthy cognition.^16^ Lower MoCA scores can provide a greater sensitivity to capture individuals who are at earlier stages of AD.^17^ On the other hand, the average annual rate of decline was not significantly different between the 2 measures, suggesting that either measure can be used to examine the clinical course of cognitive decline in patients with AD. However, each test should only be compared to itself; we found discrepancies in the rate of decline for patients with and without diabetes mellitus that were nearly significant (*P*=0.09) but opposite in directionality depending on the test. Additionally, the difference in decline for smokers vs nonsmokers was not significant across changes in MoCA scores despite being significant in MMSE scores. Tobacco smoking has long been studied as a substantial risk factor for AD development^18^; however, little research highlights its effects on increasing the actual rate of decline. Tobacco smoking is assumed to contribute to mental state decline through effects on atherosclerotic disease and oxidative stress that can exacerbate vascular dementia and neural death.^19^

Limitations to this research include differences among MMSE and MoCA test administrators. Confounding variables also include level of education, primary language, and poor vision. Additionally, some patients had cognitive impairment that was too severe for MMSE and MoCA testing and was not captured in this study. We used a cutoff score of 26 to exclude unimpaired individuals, which is sensitive and specific to indicate normal vs cognitive impairment,^17^ so this study also did not capture individuals who may have begun to develop early symptoms of cognitive impairment. Furthermore, some patients had only 2 time points available to compare MMSE and MoCA scores. With only 2 time points available, determining whether a difference in MMSE or MoCA scores is representative of a true difference from baseline cognitive state is difficult. Other confounding variables, such as time of day or even the mood status of the patient, could introduce confounding variables.

Overall, our results support previous research that suggests most variables are not significant in explaining decline rate variabilities.^7^ This study provides a useful comparison for future clinical trials to better understand the rate of cognitive decline in patients with AD at Ochsner Medical Center-New Orleans.

## CONCLUSION

AD is a devastating disease with significant impacts on morbidity, mortality, and health care costs. We describe the rate of decline and characteristics of patients with AD in urban Louisiana, data that will help serve as a baseline comparison as novel diagnostic tools and treatment modalities are implemented to reduce the impact of AD in the area.

## Notes

*This article meets the Accreditation Council for Graduate Medical Education and the American Board of Medical Specialties Maintenance of Certification competencies for Patient Care and Medical Knowledge.*
